# Survey dataset on work-life conflict of women in the construction industry

**DOI:** 10.1016/j.dib.2018.04.095

**Published:** 2018-05-01

**Authors:** Patience F. Tunji-Olayeni, Adedeji O. Afolabi, Bukola A. Adewale, Ayoola O. Fagbenle

**Affiliations:** a Covenant University, Nigeria; b Osun State College of Education, Nigeria

## Abstract

Work-life conflict can have a detrimental effect on family life, particularly for women who have to work in order to support their families financially. The data set presents the views of 50 female construction professionals in Lagos, Nigeria through a purposive sampling technique with the aid of questionnaire. Categorical Regression was used to assess the effect of work pressure on family expectations. The features of the respondents in terms of profession, years of experience, office location and household characteristics were presented in bar chart. Analysis of the data can provide information on the work experiences of women in the construction industry particularly work load, hours worked per day, work on weekends and work on holidays. The data can also provide insights on the family expectations that are significantly affected by work pressure.

**Specifications table**

**Table:** 

Subject area	*Construction*
More specific subject area	*Work - Life Conflict*
Type of data	*Tables and Figures*
How data was acquired	*Field Survey*
Data format	*Raw*
Experimental factors	*Purposive sampling of female construction professionals*
Experimental features	*Descriptive statistics and categorical regression*
Data source location	*Lagos, Nigeria*
Data accessibility	*Data is attached*

**Value of the data**

• To provide an understanding of the work experiences of women in the construction industry.
• To identify the significant family expectations affected by work pressure.
• To guide policies on reducing work-life conflict of women in the construction industry.
• The data can be modified for use in other context.

## 1 Data

**Fig. 1:**
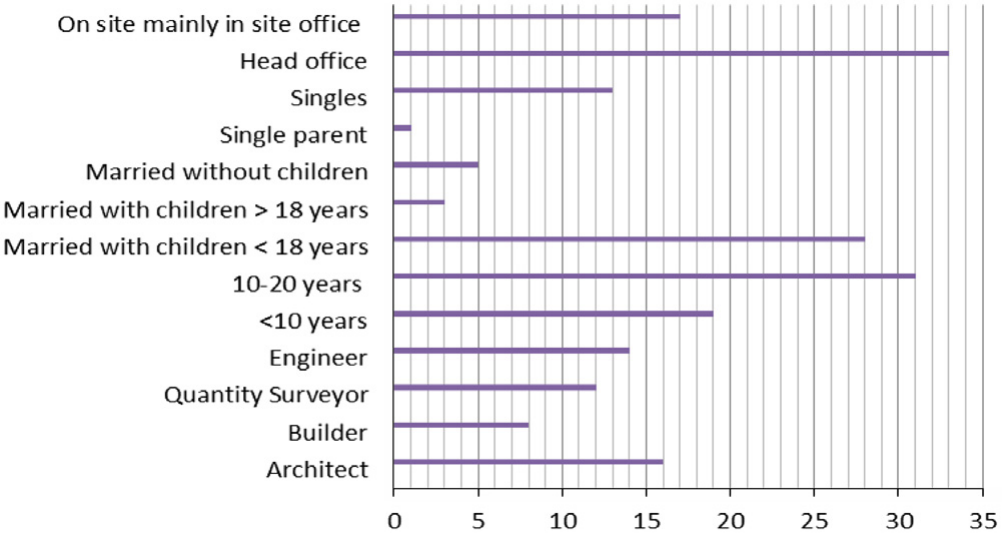
Characteristics of respondents.

**Fig. 2:**
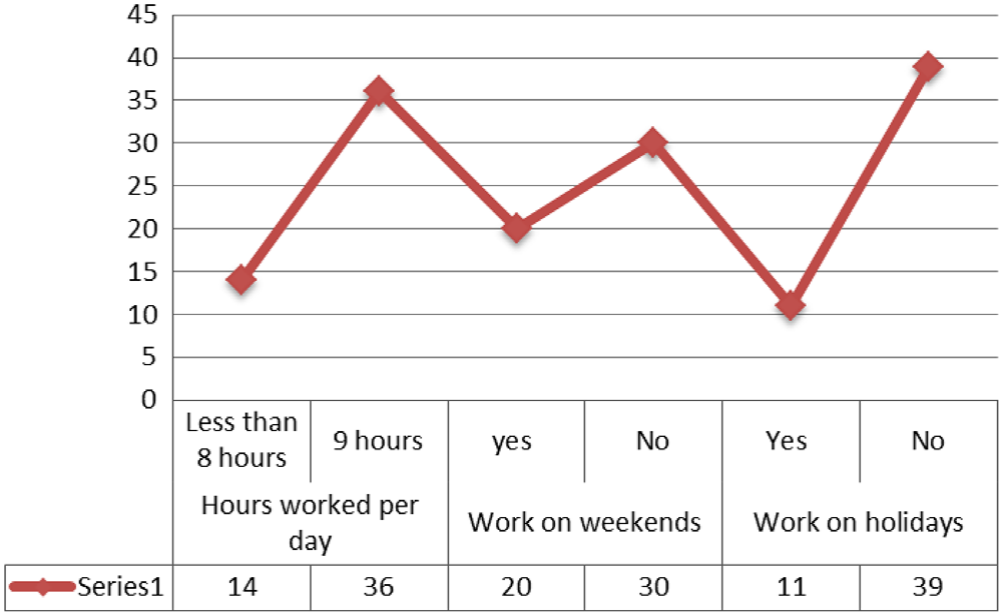
Some work experiences of women in the construction industry.

The dataset presented was obtained from women in the construction industry in Lagos, Nigeria. Ninety three questionnaires were distributed. However, only 50 of the questionnaires were returned and found suitable for analysis. The characteristics of the respondents in terms of designation, years of experience, office location and household features are shown in Fig. 1. Work experiences of the respondents focusing on hours worked per day, work on weekends and work on holidays is provided in Fig. 2. Categorical regression was used to assess the effect of work pressure on family expectations (Table 1). Table 1 shows the categorical regression (CAT REG) of work pressure affecting family expectations. The CATREG shows that work pressure affect family expectations with *R* square values of 100%. The significant factors affecting family expectations are problem with children school transportation system (88.7%), attending children׳s school event (64.3%), taking children for doctor׳s appointment (100%), spending time with family (28.6%), helping with children home work (39.7%), community participation (6%), fun time with children (10.4%), summer holidays (18.5%), house chores (1.4%) and shopping (7%). Work pressure had no impact on 4 of the family expectations. These roles included dependable children school transportation system (with 0.277>0.05), staying at home with a sick child (with 0.971>0.05), visiting acquaintances (with 0.348>0.05) and family meal time (with 0.293>0.05). The data obtained can be used to compare experiences of women construction professionals in other countries.

**Table 1:** Categorical regression of the impact of work pressure on family expectations.

	Beta	Significance
*R* Square	1.000	
*F*	1.356EA	0.000
Dependable children school transportation system	0.273	0.277
Problem with children school transportation system	0.887	0.000
Attending children׳s school event	0.643	0.000
Staying at home with a sick child	−0.066	0.971
Taking children for doctor׳s appointment	−1.389	0.000
Spending time with family	−0.286	0.000
Helping with children home work	0.397	0.000
Visiting friends	0.043	0.348
Community participation	0.006	0.053
Family meal time	0.003	0.293
Fun time with children	−0.104	0.000
Summer holidays	−0.185	0.000
House chores	−0.014	0.000
Shopping	−0.007	0.004

## 2 Experimental design, materials and methods

The data collected was based on previous work. Details of similar work on the subject can be found in Refs. [1], [2], [3], [4], [5], [6], [7], [8], [9], [10], [11], [12], [13], [14]. A total of 93 questionnaires were distributed to women construction professionals in Lagos state. Out of which 50 questionnaires were returned, representing 53.76% return rate. Purposive sampling was used to administer the questionnaire to the respondents because of the characteristics of the sample and easy access of the respondents to the researcher. The questionnaire was measured using a five point Likert scale questionnaire. The respondents comprised of women construction professionals who are Architects, Builders, Quantity Surveyors and Builders. Survey design was used because it can predict respondents׳ characteristics. Some researchers [15], [16], [17], [18], [19], [20] used survey design to achieve their research objectives.
